# Effects of Different Packaging on the Purine Content and Key Enzymes of Refrigerated Yellow Croaker (*Larimichthys crocea*)

**DOI:** 10.3390/foods14152732

**Published:** 2025-08-05

**Authors:** Tiansheng Xu, Wenxuan Lu, Bohan Chen, Dapeng Li, Jing Xie

**Affiliations:** 1 College of Food Science and Technology, Shanghai Ocean University, Shanghai 201306, China; xutiansheng44@163.com (T.X.); cbobo0429@163.com (B.C.); jxie@shou.edu.cn (J.X.); 2 Research Center of Aquatic-Product Processing & Preservation, Shanghai Ocean University, Shanghai 201306, China; 3 Key Laboratory of Aquatic Products High-Quality Utilization, Storage and Transportation (Co-Construction by Ministry and Province), Ministry of Agriculture and Rural Affairs, Shanghai Ocean University, Shanghai 201306, China

**Keywords:** large yellow croaker, purine, ATP-related compounds degradation, enzyme activities

## Abstract

In this study, we investigated the effects of air packaging, vacuum packaging and modified atmosphere packaging (CO_2_/N_2_: 80/20) on the purine metabolism and enzyme activities of refrigerated large yellow croakers. The results showed that modified atmosphere packaging significantly inhibited microbial growth, delayed adenosine triphosphate degradation and maintained higher IMP content (1.93 μmol/g on day 21) compared to the air packaging group (2.82 μmol/g on day 12). The total purine content increased with storage time, with hypoxanthine content increasing significantly and occupying most of the total content, which was the key factor for the elevation of purine, followed by adenine content showing a significant decreasing trend. Hypoxanthine accumulation was significantly suppressed in the modified atmosphere packaging group (2.31 μmol/g on day 18), which was much lower than that in the air packaging group (5.64 μmol/g), whereas xanthine and guanine did not show significant differences among the groups. The key enzymes xanthine oxidase and purine nucleoside phosphorylase were much less active in modified atmosphere packaging, effectively delaying the cascade reaction of inosine monophosphate → hypoxanthine → xanthine. The study confirmed that modified atmosphere packaging intervenes in purine metabolism through enzyme activity regulation, providing a theoretical basis for the preservation of low purine aquatic products.

## 1. Introduction

Gout is a common chronic non-communicable disease, primarily caused by purine metabolism disorder and impaired excretion of uric acid (Ua) [1]. Recent studies demonstrated that diet played a significant role in gout, in addition to genetic factors [2]. With the improvement of living standards and changes in dietary patterns, the incidence of gout in China has been gradually increasing. Researchers analyzed clinical data from 93 gout patients in China and found that the incidence of gout is higher in coastal areas than in inland areas [3], which might be related to dietary structure. Fish, shrimp, and shellfish resources were abundant in coastal areas, and the availability of these seafood resources significantly contributed to enhancing the nutritional level of local residents. The aquatic food system is facing the challenge of an accelerated transformation in order to achieve the sustainability, equity and nutritional health goals of the global fisheries and aquaculture industry [4]. This transformation not only requires attention to the environmental carrying capacity and social equity of the industry, but also to the scientific assessment of the nutritional value of aquatic products and health guidance. It has been found that excessive purine content in seafood can lead to elevated Ua levels in the human body, which ultimately induces gout [5]. Therefore, a systematic study of the purine content of marine fish and its distribution characteristics can provide consumers (especially gout sufferers) with a scientific basis for dietary choice [6,7]. At the same time, it can provide key data support for the healthy transformation of the aquatic food system and can help the industry develop in a direction that is more in line with human health needs.

The large yellow croaker (*Larimichthys crocea*), a commercially valuable marine species, had gained global recognition due to its superior organoleptic quality and exceptional nutritional profile, particularly its rich protein and lipid content [8]. However, coupled with increasing concerns about the potential health risks associated with high-purine foods, the high purine levels in large yellow croaker and its products have impeded their development. In the large yellow croaker, purines were mainly adenine (Ad), guanine (Gua), hypoxanthine (Hx) and xanthine (Xa). They primarily originated from the metabolism of nucleotides, in particular adenosine triphosphate (ATP) degradation. The degradation of ATP and its related products represented a crucial biochemical transformation during fish storage: ATP → ADP → AMP → IMP → HxR → Hx [9]. Research demonstrated that inosine 5′-monophosphate (IMP), a degradation product of ATP, served as a key indicator of fish freshness. The microbial utilization of IMP resulted in the formation of spoilage substances, which were also purines such as Hx, Xa and Ua [10,11]. The degradation of ATP and its related products was closely linked to microbial growth and reproduction, and different microorganisms could influence the degradation pathway. Modified atmosphere packaging (MAP) directly inhibits lipid and protein oxidation reactions by reducing oxygen concentration, while high concentrations of carbon dioxide inhibit the growth of aerobic bacteria, thereby reducing oxidative stress after fish slaughter [12]. In sea bass (*Lateolabrax japonicus*), modified atmosphere packaging (MAP, 60% CO_2_/40% N_2_) significantly inhibited microbial growth, delayed lipid oxidation and protein degradation, and extended the shelf life of sea bass fillets from 12 days to 18 days [13]. This is similar to the mechanism of MAP preservation of large yellow croaker in this study, both of which inhibit spoilage-related processes by regulating the gas environment. Modified atmosphere packaging directly inhibits lipid and protein oxidation reactions by reducing oxygen concentration, while high concentrations of carbon dioxide inhibit the growth of aerobic bacteria, thereby reducing oxidative stress after fish slaughter [14,15]. However, the metabolic mechanisms of purines in large yellow croaker remained incompletely systematically analyzed.

Purine catabolism was enzymatically regulated through sequential catalytic transformations [16]. Initial ATP hydrolysis to adenosine diphosphate (ADP) was mediated by ATPase (EC 3.6.1.3), followed by the catalyzed conversion of adenylate kinase (EC:2.7.4.3) to adenosine monophosphate (AMP). AMP deaminase (AMPD, EC 3.5.4.6) facilitated AMP conversion to inosine monophosphate (IMP), which underwent dephosphorylation through the coordinated actions of acid phosphatase (ACP, EC 3.5.3.2), alkaline phosphatase (ALP, EC 3.5.3.1) and 5′-nucleotidase (5′-NT, EC 3.1.3.5) to generate hypoxanthine riboside (HxR). Subsequent cleavage by purine nucleoside phosphorylase (PNP, EC 2.4.2.1) yielded Hx. Parallel GTP degradation involved enzymatic complexes producing Gua, while AMP metabolism diverged through 5′-NT-mediated adenosine formation. Adenosine underwent stepwise conversion via adenine deaminase (EC 3.5.4.2) to Hx, converging with Gua-derived pathways. Guanine deaminase (EC 3.5.4.3) and xanthine dehydrogenase/oxidase (EC 1.17.1.4/1.17.3.2) processed Gua and Hx to Xa, respectively, culminating in xanthine oxidase (XOD, EC 1.17.3.2)-catalyzed oxidation to Ua.

This study investigated the effects of different packaging methods (air packaging, vacuum packaging, and MAP) on the purine content and key enzyme activity of refrigerated large yellow croaker. The microbial growth and quality changes of large yellow croaker under different packaging conditions were examined by detecting the total viable cell count, pH, conductivity and volatile basic nitrogen. High-performance liquid chromatography was used to analyze the degradation dynamics of ATP-related compounds (ATP, IMP, Hx, etc.) and changes in purine (Ad, Gua, Hx, Xa) content in large yellow croaker during different storage periods. Additionally, the activity of key purine metabolism enzymes (XOD, PNP, 5′-NT, etc.) was measured to investigate the regulatory effects of different packaging methods on the purine metabolism cascade reactions. This study aimed to reveal the effects of different packaging methods on purine metabolism in large yellow croaker and the regulatory mechanisms of enzyme activity. Based on the results, the regulatory patterns of different packaging methods on the purine metabolism pathway (IMP → Hx → Xa) in large yellow croaker were obtained, and the extent of the role of key enzymes in this process was analyzed. This study laid the foundation for understanding the mechanism of purine metabolism in large yellow croaker under refrigerated conditions.

## 2. Materials and Methods

### 2.1. Fish Sample Preparation

Large yellow croaker (*Larimichthys crocea*; weight 0.63 ± 0.21 kg, length 35.5 ± 3.7 cm) were purchased live from a local fresh market in Shanghai, China. The fish were transported to the laboratory in oxygenated clean water. Upon arrival, they were scaled, gutted, skinned, cleaned, and immediately cut into four similarly sized fillets (40 ± 5 g). The fillets were wiped with clean kitchen paper and packed individually in high-density polyethylene (HDPE) plastic bags. The individually packed fillets were then randomly divided into three groups for packaging at 4 °C according to the following treatments: air packaging (AP), vacuum packaging (VP) and modified atmosphere packaging (MAP, CO_2_/N_2_: 80/20) [17]. Biochemical analysis and microbiological testing were performed every three days on randomly selected fillets (*n* = 3 per group per time point).

### 2.2. Total Viable Count (TVC)

Total viable count (TVC) was determined according to a method modified from other literature [18]. Take 5 g of fish meat and homogenize it with 45 mL of sterile 0.9% saline solution. After gradient dilution, take 100 μL and spread it on a PCA plate. Incubate at 30 °C for 48 h and count. TVC is expressed as log10 CFU/g.

### 2.3. The pH and Electrical Conductance (EC)

After weighing 2 g of dorsal muscle from the large yellow croaker and mixing it with 18 mL of distilled water, the mixture was homogenized and then centrifuged at 4 °C for 10 min at 6180× *g*. The supernatant was removed to measure the pH using a pH meter (BPH-7100 BELL, Analytical Instruments (Dalian) Co., Ltd., Dalian, China) (*n* = 3), and the EC values were measured with an EC meter (C-6800 BELL, Analytical Instruments (Dalian) Co., Ltd., Dalian, China) (*n* = 3).

### 2.4. Total Volatile Basic Nitrogen (TVB-N)

Total volatile basic nitrogen was determined according to Yan’s method [17] using a Kjeltec nitrogen meter (Kjeltec 8400, Foss, Hillerød, Denmark), and the results were expressed as mgN/100 g of meat (*n* = 3).

### 2.5. Determination of ATP-Related Compounds

The evaluation of ATP-related compounds was carried out in strict accordance with the methodological framework established by Chen et al. [18]. The concentrations of ATP, ADP, AMP, IMP, HxR and Hx were quantified through HPLC analysis following established extraction protocols. Briefly, 5 g aliquots of fish tissue were homogenized with 10 mL of chilled 10% perchloric acid using a HR-500 homogenizer (Shanghai, China). After initial centrifugation (8000× *g*, 10 min, 4 °C), the resulting pellet underwent sequential re-extraction with 10 mL of chilled 5% PCA through secondary homogenization and equivalent centrifugation parameters. Combined supernatants were neutralized to pH 6.5 using 10 mM and 1 mM KOH solutions, then volumetrically adjusted to 50 mL in polypropylene centrifuge tubes. The prepared extracts were membrane-filtered (0.22 μm) prior to chromatographic separation using a Waters E2695 HPLC system (Milford, MA, USA) equipped with a COSMOSIL 5C18-PAQ analytical column (4.6 mm × 250 mm). At a flow rate of 1 mL/min, phosphate buffer solution (0.05 mol/L, pH 6.5) was used as the eluent, and isocratic elution was performed at a detection wavelength of 254 nm. Quantitative analysis was achieved through external calibration with certified reference standards for each target analyte.

### 2.6. Determination of Purines

#### 2.6.1. Preparation of Purine Standard Solutions

A total of 0.1 g of each of the four purine standards (Ad, Gu, Hx and Xa) was accurately weighed, 5 mL of 0.1 mol/L NaOH solution was added to promote dissolution, and then the volume was fixed to 1000 mL with ultrapure water to prepare a 0.1 g/L purine standard solution, which was stored in a 4 °C freezer.

#### 2.6.2. Sample Preparation

The acid hydrolysis method of Krata et al. [19] was adopted, with appropriate adjustments. The experiment was done three times in parallel. After the fish has been stirred, 0.2 g of minced flesh was weighed and put into a 20 mL plugged test tube. A total of 3 mL of perchloric acid with a volume fraction of 10% was added. The mixture was hydrolyzed in a boiling water bath for 60 min, and then quickly cooled in an ice bath. The pH of the hydrolysate was adjusted to 3.8 with 1 mol/L KOH solution and was fixed to 5.0 mL. Then they were centrifuged at 3000 r/min for 30 min, and the supernatant was filtered through a 0.45 μm filter and stored in a 4 °C freezer for later use.

#### 2.6.3. Optimization of Detection Methods for Purine Components

The content of purine components was determined by HPLC. Due to the significant influence of mobile phase pH on the detection results, a 0.02 mol/L KH_2_PO_4_ buffer with pH of 3.6, 3.8 and 4.0 was set as the mobile phase for comparative experiments to obtain the optimal HPLC determination conditions. High-performance liquid chromatography (Waters E2695, Milford, MA, USA) with a COSMOSIL 5C18-PAQ Column (4.6 mm × 250 mm) was used at a flow rate of 1.0 mL/min; column temperature 30 °C; injection volume: 20 μL; and ultraviolet wavelength 254 nm.

### 2.7. Determination of Enzyme Activities (ACP, AKP, 5′-NT, PNP, and XOD)

Enzymatic activities in fish tissues were quantified through standardized assay protocols. Tissue homogenates were prepared at 1:9 (*w*/*v*) ratio using chilled 0.9% NaCl solution, followed by centrifugation (2500× *g*, 10 min) to obtain clarified supernatants. Enzyme analyses were performed using commercial kits: acid phosphatase (ACP), alkaline phosphatase (AKP), 5′-nucleotidase (5′-NT) and XOD activities determined with respective kits (Nanjing Jiancheng Institute of Biological Engineering, Nanjing, China). PNP activity was measured using a species-specific ELISA kit (COIBO Biotechnology, Shanghai, China) Total protein quantification employed the biuret method (Nanjing Jiancheng Bioengineering Institute, Nanjing, China), with absorbance measured at 640 nm after 30 min incubation (25 °C) of 1 mL supernatant mixed with 4 mL chromogenic reagent [20].

### 2.8. Statistical Analysis

Experimental analyses were conducted in triplicate with statistical significance evaluation performed in IBM SPSS Statistics 27 (SPSS Inc., Chicago, IL, USA). Quantitative results were presented as mean ± SD, with graphical visualizations generated through Origin 2021 (SPSS Inc., Chicago, IL, USA). Datasets underwent one-way ANOVA with Duncan’s post-hoc test (α = 0.05) for comparative analysis.

## 3. Results

### 3.1. TVC

**Figure 1:**
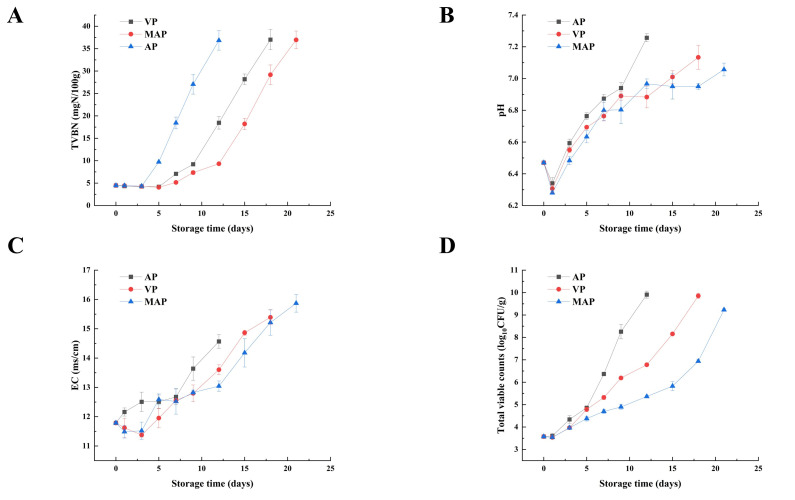
Changes in TVC (**A**), pH (**B**), EC (**C**), TVBN (**D**) in each packing group during refrigeration of large yellow croaker fillets (AP: air packaging, VP: vacuum packaging and MAP: modified atmosphere packaging (CO_2_/N_2_:80/20)).

In the study of fish preservation, the degree of microbial reproduction intuitively showed the shelf life and quality trend of fish meat. The TVC variation of large yellow croaker fillets was shown in Figure 1A. The initial low count of the TVC (3.5 log10 CFU/g) indicated that the quality of the fish was fresh. During the nine days of storage, the TVC of large yellow croaker fillets increased rapidly to 7.9 log10 CFU/g, which means that the quality of large yellow croaker fillets in the AP group became unacceptable on day 9. The VP group became unacceptable at 15 days with a TVC of 8.1 log10 CFU/g. The MAP group showed high levels of bacterial inhibition, and their TVC exceeded 7 log10 CFU/g on day 18, consistent with Yan’s study [17].

### 3.2. The pH and EC

The pH level was intrinsically linked to endogenous enzymes and microbial proliferation [20]. During storage, the pH of fish fillets exhibited a pattern of initial decline followed by a subsequent rise in Figure 1B. Initially, at a pH of 6.47, all samples experienced a decrease within the first day, with the MAP condition reaching the lowest pH of 6.28. Starting from the third day onwards, the pH in all groups began to climb, with the AP and VP groups showing a sharp increase, while the MAP group’s pH rose more gradually. Figure 1B illustrated that MAP significantly retarded the pH increase in fillets (*p* < 0.05).

Figure 1C shows that the initial electrical conductivity (EC) was 11.78 mS/cm, with significant differences (*p* < 0.05) among the groups early in storage. The EC values of the AP and VP groups rose quickly, with the AP group’s rate of increase notably surpassing that of the other treatment groups. In the later stages of storage, the EC values for AP, VP and MAP samples were 14.57 mS/cm, 15.39 mS/cm and 15.87 mS/cm, respectively.

### 3.3. TVB-N

Total volatile basic nitrogen refers to the alkaline nitrogen-containing substances such as ammonia and amines produced by the decomposition of proteins in animal foods due to the action of enzymes and bacteria in the process of spoilage [21]. According to Figure 1D, all three groups show a trend of stabilization followed by a continuous increase. The initial value of fresh large yellow croaker fillets was about 4.47 mg N/100 g. The sample value changed little in the first three days (*p* > 0.05), and the quality of the fish was better at this time. This was similar to the study that showed that there was no significant difference in TVB-N at the beginning of storage [21]. The spoilage rate of large yellow croaker fillets in the AP group was faster, reaching 36.83 mg N/100 g on the ninth day, exceeding the maximum allowable level for marine fish (35 mg N/100 g) [22]. The VP group of large yellow croaker fillets reached an imperceptible value (37.00 mg N/100 g) at 18 days. Similar to the results for TVC, MAP showed the best freshness retention, exceeding the limit (36.96 mg N/100 g) at 21 days. The results showed that the MAP group effectively inhibited the production of TVB-N (*p* < 0.05), which was the same as the change in TVC in most studies [17].

### 3.4. Changes in ATP-Related Products

**Figure 2:**
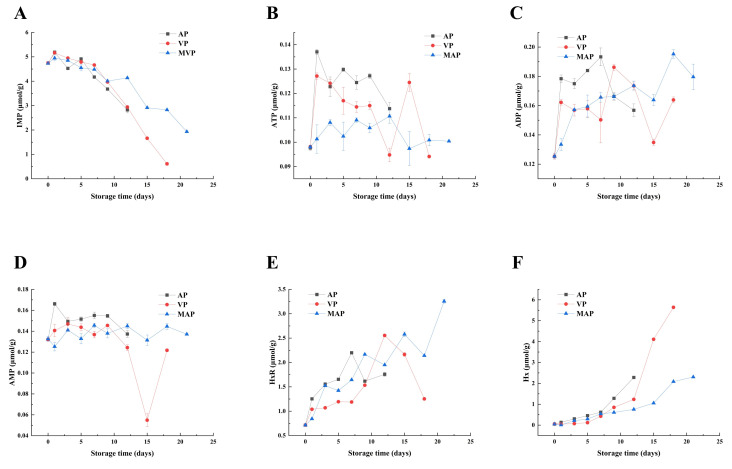
Changes in IMP (**A**), ATP (**B**), ADP (**C**), AMP (**D**), HxR (**E**) and Hx (**F**) in each packing group during refrigeration of large yellow croaker fillets (AP: air packaging, VP: vacuum packaging and MAP: modified atmosphere packaging (CO_2_/N_2_:80/20)).

ATP-related products in fish samples under different packaging conditions showed differential degradation regularity (Figure 2A–F). ATP, ADP and AMP declined rapidly on the first day of storage (Figure 2B–D), of which ADP and AMP, as intermediates in the degradation of ATP, were maintained at a low level throughout the storage period (0.11 μmol/g on the 12th day in the AP group). The degradation rate of IMP did not differ significantly among groups in the first five days of storage (Figure 2A), and began to decrease significantly after five days, with the largest decrease in the AP group (2.82 μmol/g on day 12), and the MAP group decreased to 1.93 μmol/g on day 21. The HxR content in the AP group was initially 0.71 μmol/g, which increased and then decreased to 1.75 μmol/g during storage. The VP group similarly increased slowly and then fluctuated and decreased to 1.25 μmol/g, whereas the MAP group basically showed an increasing trend, although there was a downward fluctuation that reached a peak of 3.25 μmol/g at 21 days (Figure 2E). During the initial storage period, the Hx value of the AP group was 0.05 μmol/g, steadily increasing over the first seven days, and then sharply rising to 2.28 μmol/g on day 12; The VP group showed a similar trend to the AP group, with a continuous increase over the first 15 days, followed by a sharp rise on day 15, reaching 5.64 μmol/g on day 18. The MAP group began to rise sharply on the 15th day, reaching 2.08 μmol/g on the 18th day, and then slowly increased to 2.31 μmol/g on the 21st day (Figure 2F).

### 3.5. Changes in Purines

**Figure 3:**
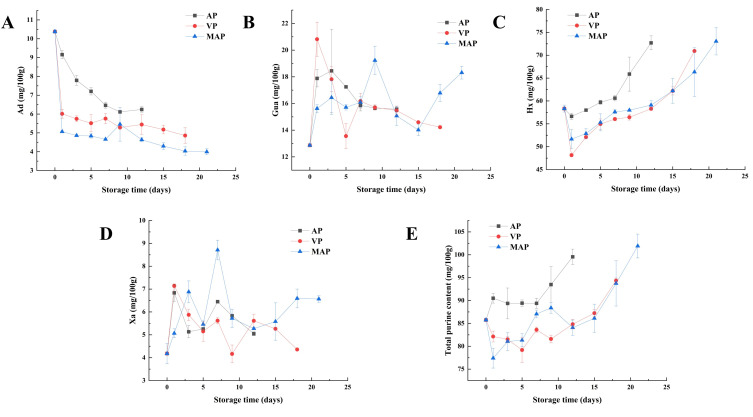
Changes in Ad (**A**), Gua (**B**), Hx (**C**), Xa (**D**) and total purine content (**E**) in each packing group during refrigeration of large yellow croaker fillets (AP: air packaging, VP: vacuum packaging and MAP: modified atmosphere packaging (CO_2_/N_2_:80/20)).

Purine metabolites (Ad, Gua, Hx and Xa) exhibited distinct dynamic patterns during cold storage of yellow croaker under AP, VP and MAP (Figure 3A–E). Ad levels declined continuously in the AP group, decreasing from 10.48 mg/100 g (day 0) to 6.08 mg/100 g (day 12). In the VP group, Ad remained stable initially (10.48–7.34 mg/100 g, day 0–6) before a sharp drop to 5.24 mg/100 g (day 9), followed by fluctuations. The MAP group showed a gradual decrease over the entire storage period, reaching 4.16 mg/100 g by day 21. In the AP group, Gua followed a “rise-fall” pattern: it peaked at 17.58 mg/100 g on day 1 (day 0: 12.92 mg/100 g) and then declined, stabilizing at 15.48 mg/100 g by day 12. The VP group shows significant fluctuations: a peak of 19.05 mg/100 g on day 1, a decline to 14.50 mg/100 g by day 12, a trough at 13.62 mg/100 g on day 15 and a final rise to 17.51 mg/100 g by day 21. The MAP group showed slow degradation until day 12 (14.23 mg/100 g) before late-stage accumulation, reaching 18.37 mg/100 g by day 21. Hx increased consistently across groups. In the AP group, Hx remained stable in the first six days (57.51–57.01 mg/100 g) before accelerating: 59.22 mg/100 g (day 9) and 62.48 mg/100 g (day 12). The VP group showed a transient trough at 54.33 mg/100 g (day 7), followed by recovery to 57.16 mg/100 g (day 12) and rapid accumulation to 71.21 mg/100 g (day 18). The MAP group accumulated steadily, reaching 73.50 mg/100 g by day 21 (day 12: 59.08 mg/100 g). Xa content changed minimally. The AP group fluctuated: peaking at 7.08 mg/100 g (day 1) and declining to 5.13 mg/100 g (day 12). The VP group increased to 8.95 mg/100 g (day 12) before dropping to 3.97 mg/100 g (day 18). The MAP group remained stable in the early stage (4.54–5.33 mg/100 g, day 0–12) and later stabilized at 4.89–6.69 mg/100 g (day 15–21). Total purine (sum of Ad, Gua, Hx and Xa) increased across all groups. The AP group peaked at 91.14 mg/100 g (day 1) and fluctuated to 99.81 mg/100 g by day 18 (sampling terminated afterward). The VP group troughing at 79.01 mg/100 g (day 6), then rose to 95.82 mg/100 g (day 12) and stabilized at 94.85 mg/100 g (day 18). The MAP group showed slow early accumulation (85.44–87.19 mg/100 g, day 0–7) but rapid late growth, reaching 102.71 mg/100 g by day 21.

### 3.6. Changes in Key Enzymes

**Figure 4:**
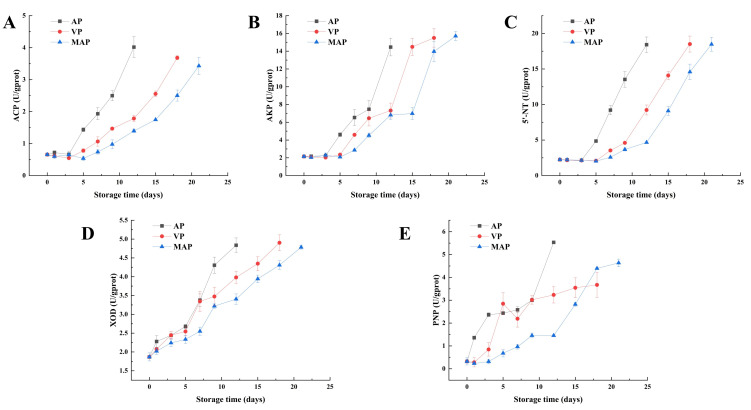
Changes in ACP (**A**), AKP (**B**), 5′-NT (**C**), XOD (**D**) and PNP (**E**) in each packing group during refrigeration of large yellow croaker fillets (AP: air packaging, VP: vacuum packaging and MAP: modified atmosphere packaging (CO_2_/N_2_:80/20)).

Key enzyme activities showed package-dependent dynamic changes during cold storage of large yellow croaker fillets (Figure 4A–E). The initial activities of ACP and AKP were 0.66 U/gprot and 2.14 U/gprot, respectively, and remained stable for the first three days of storage. On day 5, the ACP activity in the AP group (1.58 U/gprot) was significantly higher than that in the VP (1.02 U/gprot) and MAP (0.89 U/gprot) groups, followed by a rapid increase in the ACP activities of the VP and MAP groups from day 5 to 12 (average daily increase >0.15 U/gprot). The activity of 5′-Nucleotidase (5′-NT) in the AP group reached 4.86 U/gprot on day 5 and then increased continuously to 18.42 U/gprot on day 12. The accelerated increase in activity in the AP group was significantly earlier than that in the VP and MAP groups: in the VP group the increase was slow for the first nine days and reached 18.5 U/gprot on day 18, while in the MAP group the increase was also slow for the first 12 days and rose to 18.48 U/gprot on day 21. PNP activity increased sharply (182% increase) in the AP group for the first five days, then slowed down, and then increased rapidly to 5.53 U/gprot on day 12; in the VP group, PNP increased rapidly for the first five days, and then slowly increased to 3.67 U/gprot; and in the MAP group, PNP activity increased in the late storage period (12–21 days), then continued to increase to 4.63 U/gprot. XOD activity was significantly elevated at the end of storage, reaching 4.84, 4.90 and 4.78 U/gprot in the AP, VP and MAP groups, respectively.

## 4. Discussion

In this study, the effects of different packaging on the refrigerated quality of large yellow croaker fillets were systematically evaluated by TVC, pH, EC and TVB-N. The initial total viable count (TVC) of fresh fillets was 3.5 log_10_ CFU/g, indicating good initial freshness. TVC data showed that the AP group reached unacceptable levels (7.9 log10 CFU/g) on day 9, while the VP and MAP groups were delayed until day 15 (8.1 log10 CFU/g) and day 21 (>7 log10 CFU/g) [23], respectively, indicating that MAP may inhibited the proliferation of spoilage bacteria (e.g., *Pseudomonas* spp.) due to high concentration of CO_2_ (80%) (Figure 1A). CO_2_ is an important gas with bacteriostatic effect in MAP. High concentration of CO_2_ can increase the solubility of CO_2_ and retard microbial growth, and N_2_ is an inert gas that makes the packaging material less susceptible to collapse [24]. Previous studies have shown that CO_2_/N_2_ gas mixtures have similar bacteriostatic and shelf-life extension effects on aquatic products such as salmon and cod, similar to the results of this experiment [25]. The initial decrease in pH (6.47 → 6.28 on day 1 in the AP group) was associated with the accumulation of inorganic phosphate and lactate produced by the degradation of ATP, whereas the late increase originated from the microbial metabolism of alkali production (e.g., ammonia-like substances) and autolysis [26]. The rapid increase in EC (14.57 mS/cm on day 21 in the AP group) reflected increased ionic leakage from the fillets, whereas the slower increase in EC in the MAP group (15.87 mS/cm on day 21) indicated that the modified atmosphere packaging inhibited the production of small molecules in the fillets and suppressed the rate of spoilage, thus slowing down the deterioration rate of the fillets [27,28,29]. On day 9 of the AP group, the content of TVB-N reached 36.83 mg N/100 g and its accumulation trend was directly related to microbial growth, while the later increase in pH (pH of 6.92 on day 9 of the AP group) was related to the accumulation of ammonia-based alkaline metabolites. In conclusion, MAP significantly retarded quality deterioration by modulating gas composition (CO_2_/N_2_:80/20). The mechanism involved may be that CO_2_ leads to metabolic dysfunction and growth inhibition in Pseudomonas spp. through inhibition of energy metabolic pathways (glycolysis, TCA cycle, electron transport chain) and disruption of the cell membrane [30].

**Figure 5:**
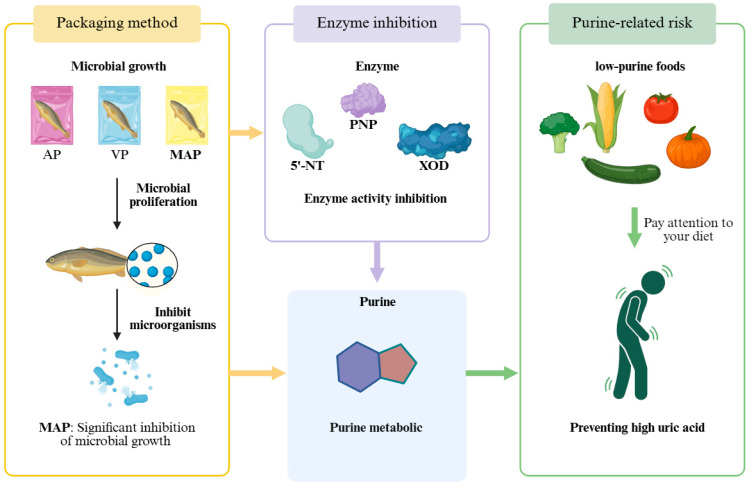
Conceptual diagram showing the relationship between refrigerated large yellow croaker (*Larimichthys crocea*) in different packaging, purine metabolism and health.

The degradation pattern of ATP-related products revealed the regulatory effect of packaging technology on nucleotide metabolism network (Figure 2). IMP, as a key intermediate product of ATP degradation, had an initial content of approximately 4.74 μmol/g in fresh fillets, a key freshness indicator contributing to the characteristic umami flavor. The change of its content (2.82 μmol/g on day 12 of the AP group vs. 1.93 μmol/g on day 21 of the MAP group) was directly correlated with microbial activity. In the early stage of storage (0–5 days), endogenous phosphatases (e.g., 5′-NT) dominated IMP degradation, whereas in the middle and late stages (>5 days) extracellular enzymes secreted by specific spoilage organisms (SSOs) accelerated the IMP → HxR conversion, a process that was particularly significant in the AP group [31]. The initial Hx content in the AP group was 0.05 μmol/g. The pattern of accumulation of Hx (2.28 μmol/g on day 12 in the AP group vs. 3.25 μmol/g on day 21 in the MAP group) was directly correlated with microbial activity (0.01 μmol/g). Moreover, Figure 3 showed a fluctuating characteristic: the generation of Ad catalyzed by endogenous adenosine deaminase in the early phase (0–7 days) and the microbial PNP-mediated HxR catabolism in the late phase (>7 days). As shown in Figure 5, we eat low-purine foods to prevent gout. In this experiment, we used different packaging methods to reduce purine production. It was worth noting that Hx, through further oxidation, generates Xa and Ua, which is directly related to the risk of hyperuricemia, making Hx a key substance to be highlighted in purine metabolism [32]. Clinically, hyperuricemia is diagnosed when human serum uric acid concentration exceeds approximately 420 μmol/L (7 mg/dL), and excessive intake of purine-rich foods (such as fish with high Hx content) is a key factor contributing to elevated uric acid levels. Therefore, doctors recommend consuming foods with low purine content, not exceeding 50 mg/100 g [33]. In the MAP group, the final content of Hx (3.25 μmol/g) reflects the regulatory effect of packaging on purine accumulation, highlighting its significance in evaluating the health-related quality of refrigerated yellow croaker. For foods with moderate to high purine content, such as red snapper, it is recommended to use MAP (CO_2_/N_2_ = 80/20) for refrigeration, which can extend the shelf life to 21 days and is more suitable for patients with hyperuricemia and gout.

The initial ACP activity of fresh fillets was 0.66 U/gprot. ACP activity in the AP group was significantly elevated on day 5 (1.58 U/gprot vs. 0.89 U/gprot in the MAP group). During the late storage period (5–12 days), the rapid increase in ACP activity in VP and MAP groups (mean daily increase >0.15 U/gprot) can be attributed to the regulatory role of packaging on microbial–enzymatic interactions. Unlike the AP group with unrestricted microbial growth, the delayed microbial proliferation in VP and MAP (Figure 1A) led to a later but accelerated release of microbial-derived phosphatases, as microbial metabolism gradually dominates phosphatase-mediated reactions (e.g., AMP dephosphorylation to adenosine) in the mid-storage stage [34]. The similar trend of AKP activity to ACP further confirms the coordinated regulation of phosphatase systems by packaging conditions, with higher activity in the AP group reflecting the stronger microbial-driven hydrolytic capacity under aerobic conditions. The earlier surge of 5′-NT activity in the AP group (peaking at 4.86 U/gprot on day 5) compared to the delayed increase in the MAP group (rising to 18.48 U/gprot after day 12) stems from the critical role of spoilage microbiota. The AP group’s TVC exceeding 7.9 log10 CFU/g on day 9 (Figure 1A) promoted massive secretion of extracellular 5′-NT by specific spoilage organisms like *Shewanella putrefaciens* [35], which directly accelerates IMP catabolism (Figure 4C). In contrast, the high CO_2_ in MAP inhibits microbial metabolism and extracellular enzyme secretion, thereby delaying the activation of 5′-NT, which explains the slower IMP degradation and better freshness retention in the MAP group (Figure 2A). The inhibitory effect of CO_2_ in the MAP group reduces microbial-derived enzyme release, thereby delaying the rise in 5′-NT activity [36]. A similar trend in the activities of ACP, AKP, and 5′-NT may be due to the continuous degradation of AMP, IMP and guanosine monophosphate (GMP) as substrates. XOD is mainly produced by microorganisms and catalyzes the conversion of Hx to Xa and then to Ua. XOD has a strong effect on purine content. In summary, the high level of Hx content directly affects the total purine content.

The change of Hx may have promoted the generation of HxR (Figure 2E), which provided a sufficient substrate for PNP and drove the surge in its activity, whereas CO_2_ in the MAP group indirectly limited the catalytic efficiency of PNP by inhibiting the metabolism of aerobic bacteria and reducing the accumulation of HxR [37]. This phenomenon may be attributed to the lower Hx accumulation in the MAP group in the early stage (2.31 μmol/g vs. 5.64 μmol/g in the AP group), which directly reduces the amount of substrate for the catalytic production of Xa by XOD. The total purine content was increasing during storage, and it was worth noting the changes of Ad and Hx. The content of Ad was decreasing, but the content of Hx was increasing, and the content of Hx occupied most of the purine content. In addition, Gua and Xa did not change significantly, and Xa increased in the late stage of storage, which may be due to the increase in Hx content and the increase in XOD and PNP activities. Therefore, the most important aspect of purines is the change in Hx, and Hx synthesis and catabolism are the key determinants of purine content. The XOD and PNP involved in this pathway become the key enzymes. PNP itself is produced by the aquatic product and the microorganisms [38]. The AP group showed a surge in PNP activity of 182% on day 5 (Figure 4E) and only a small increase (4.63 U/gprot) at the later stage in the MAP group. MAP regulates PNP activity by inhibiting microbial growth and reducing purine production, confirming its effectiveness in regulating purine metabolism. In conclusion, limiting the growth of microorganisms can limit the changes in key enzyme activities, thus limiting the production of purine substances, which provides a scientific basis and technical support for the research and development and production of low purine foods.

## 5. Conclusions

This study systematically revealed the effects of packaging technology on purine content and key enzyme activities in refrigerated yellow croaker. The MAP group significantly inhibited microbial proliferation through high concentration of CO_2_, delayed the rise of pH and the growth rate of conductivity and effectively inhibited the generation of TVB-N. The MAP group significantly retained the IMP content and inhibited the accumulation of Hx. Total purine content increased with storage time, with the highest contribution of Hx and a significant decrease in Ad. MAP regulated the IMP → Hx → Xa metabolic pathway by inhibiting the activities of 5′-NT, XOD and PNP, and ultimately realized the regulation of low purine metabolism. Future studies could focus on the conformational relationship between enzymes and proteins, such as competition for inhibition of the PNPase active site to reduce purine content and the development of targeted preservation strategies based on inhibitors of enzyme activity to achieve precise regulation of purine metabolic pathways. These results provided a theoretical basis for the development of low purine aquatic products. Practically, from an industrial standpoint, MAP (CO_2_/N_2_ = 80/20) is a preferred packaging for large yellow croaker and similar high-purine fish supply chains, balancing shelf-life and purine control to meet industrial and consumer health needs.

## Data Availability

The original contributions presented in the study are included in the article; further inquiries can be directed to the corresponding author.

## Abbreviations

5′-NT: 5′-Nucleotidase; ACP: Acid Phosphatase; Ad: Adenine; ADP: Adenosine Diphosphate; AKP: Alkaline Phosphatase; AMP: Adenosine Monophosphate; AMPD: AMP Deaminase; AP: Air Packaging; ATP: Adenosine Triphosphate; CFU: Colony Forming Units; EC: Electrical Conductance; Gua: Guanine; HDPE: High-Density Polyethylene; HPLC: High-Performance Liquid Chromatography; Hx: Hypoxanthine; HxR: Hypoxanthine Riboside; IMP: Inosine Monophosphate; MAP: Modified Atmosphere Packaging; PCA: Plate Count Agar; PNP: Purine Nucleoside Phosphorylase; SD: Standard Deviation; SSOs: Specific Spoilage Organisms; TCA: Tricarboxylic Acid Cycle; TVC: Total Viable Count; TVB-N: Total Volatile Basic Nitrogen; Ua: Uric Acid; VP: Vacuum Packaging; Xa: Xanthine; XOD: Xanthine Oxidase.

## References

[1] Zhu B., Wang Y., Zhou W., Jin S., Shen Z., Zhang H., Zhang X., Ding X., Li Y. (2022). Trend dynamics of gout prevalence among the Chinese population, 1990–2019: A joinpoint and age-period-cohort analysis. Front. Public Health.

[2] Zhang Y., Chen S., Yuan M., Xu Y., Xu H. (2022). Gout and Diet: A Comprehensive Review of Mechanisms and Management. Nutrients.

[3] Ji A., Tian Z., Shi Y., Takei R., Chang S.-J., Yip R.M.L., Yin H., Li C. (2025). Gout in China. Gout Urate Crystal Depos. Disord..

[4] FAO (2024). The State of World Fisheries and Aquaculture 2024–Blue Transformation in Action.

[5] Kaneko K., Aoyagi Y., Fukuuchi T., Inazawa K., Yamaoka N. (2014). Total Purine and Purine Base Content of Common Foodstuffs for Facilitating Nutritional Therapy for Gout and Hyperuricemia. Biol. Pharm. Bull..

[6] Jantasaeng O., Duclos M.J., Thumanu K., Okrathok S., Khempaka S. (2025). Effects of low-purine diet supplemented with *Sida acuta* Burm. f. on growth performance, purine deposition, and biomolecules in slow-growing chickens. Anim. Biosci..

[7] Sabolová M., Kulma M., Petříčková D., Kletečková K., Kouřimská L. (2023). Changes in purine and uric acid content in edible insects during culinary processing. Food Chem..

[8] Ma R., Meng Y., Zhang W., Mai K. (2020). Comparative study on the organoleptic quality of wild and farmed large yellow croaker (*Larimichthys crocea*). J. Oceanol. Limnol..

[9] Hong H., Regenstein J.M., Luo Y. (2017). The Importance of ATP-related Compounds for the Freshness and Flavor of Post-mortem Fish and Shellfish Muscle: A Review. Crit. Rev. Food Sci. Nutr..

[10] Li J., Zhou G., Xue P., Dong X., Xia Y., Regenstein J., Du M., Sun L. (2021). Spoilage microbes’ effect on freshness and IMP degradation in sturgeon fillets during chilled storage. Food Biosci..

[11] Chen B., Xu T., Yan Q., Karsli B., Li D., Xie J. (2025). Effect of temperature fluctuations on large yellow croaker fillets (*Larimichthys crocea*) in cold chain logistics: A microbiological and metabolomic analysis. J. Food Eng..

[12] Zhou Q., Li P., Fang S., Mei J., Xie J. (2020). Preservative Effects of Gelatin Active Coating Containing Eugenol and Higher CO_2_ Concentration Modified Atmosphere Packaging on Chinese Sea bass (*Lateolabrax maculatus*) during Superchilling (−0.9 °C) Storage. Molecules.

[13] Zhang X., Pan C., Chen S., Xue Y., Wang Y., Wu Y. (2022). Effects of Modified Atmosphere Packaging with Different Gas Ratios on the Quality Changes of Golden Pompano (*Trachinotus ovatus*) Fillets during Superchilling Storage. Foods.

[14] Babic Milijasevic J., Milijasevic M., Lilic S., Djinovic-Stojanovic J., Nastasijevic I., Geric T. (2023). Effect of Vacuum and Modified Atmosphere Packaging on the Shelf Life and Quality of Gutted Rainbow Trout (*Oncorhynchus mykiss*) during Refrigerated Storage. Foods.

[15] Ramadhan A.H., Yu D., Hlaing K.S.S., Jiang Q., Xu Y., Xia W. (2025). Effects of packaging and storage time on lipid and protein oxidation and modifications in texture characteristics of refrigerated grass carp (*Ctenopharyngodon idellus*) fish muscles. Int. J. Food Sci. Technol..

[16] Feng S., Wu S., Xie F., Yang C.S., Shao P. (2022). Natural compounds lower uric acid levels and hyperuricemia: Molecular mechanisms and prospective. Trends Food Sci. Technol..

[17] Yan Q., Guo M., Chen B., Zhang C., Li D., Xie J. (2023). Molecular characterization of spoilage microbiota in high CO_2_ refrigerated large yellow croaker (*Larimichthys crocea*) fillets using metagenomic and metabolomic approaches. Food Biosci..

[18] Chen B., Xu T., Yan Q., Li D., Xie J. (2024). Specific spoilage bacteria in cold chain logistics: A study on ATP-related compounds degradation in large yellow croaker (*Larimichthys crocea*). Food Biosci..

[19] Krata A.A., Domagała J., Głowacki R. (2024). Hydrophilic interaction liquid chromatography based method for simultaneous determination of purines and their derivatives in food spices. Food Chem..

[20] Li P., Mei J., Xie J. (2024). Antibacterial mechanism of CO_2_ combined with low temperature against *Shewanella putrefaciens* by biochemical and metabolomics analysis. Food Chem..

[21] Kimbuathong N., Leelaphiwat P., Harnkarnsujarit N. (2020). Inhibition of melanosis and microbial growth in Pacific white shrimp (*Litopenaeus vannamei*) using high CO_2_ modified atmosphere packaging. Food Chem..

[22] Li X., Zheng S., Wu G. (2020). Nutrition and metabolism of glutamate and glutamine in fish. Amino Acids..

[23] Chen B., Yan Q., Li D., Xie J. (2025). Degradation mechanism and development of detection technologies of ATP-related compounds in aquatic products: Recent advances and remaining challenges. Crit. Rev. Food Sci. Nutr..

[24] Yavuzer E., Kose M. (2022). Prediction of fish quality level with machine learning. Int. J. Food Sci. Technol..

[25] Park E.J., Jung S.Y., Kim S.C., Lee D.S., An D.S. (2023). Modified atmosphere packaging of flounder fillet: Modelling of package conditions and comparison of different flushing atmospheres for quality preservation. Int. Food Res. J..

[26] Chan S.S., Rotabakk B.T., Løvdal T., Sone I., Roth B. (2025). Sub-chilling methods for Atlantic salmon with 7 days in refrigerated seawater and subsequent sub-chilled storage. Sci Rep..

[27] Xu T., Yan Q., Chen B., Li D., Xie J. (2025). Novel perspective on spoilage metabolism of refrigerated large yellow croaker (*Larimichthys crocea*) under air-packaging and modified atmosphere-packaging: Insights into adenosine triphosphate degradation. Food Sci. Hum. Wellness.

[28] Almeida C., Neves M.C., Freire M.G. (2021). Towards the use of adsorption methods for the removal of purines from beer. Molecules.

[29] Amaral R.A., Pinto C.A., Lima V., Tavares J., Martins A.P., Fidalgo L.G., Silva A.M., Gil M.M., Teixeira P., Barbosa J. (2021). Chemical-Based Methodologies to Extend the Shelf Life of Fresh Fish—A Review. Foods.

[30] Gopi N., Rekha R., Vijayakumar S., Liu G., Monserrat J.M., Faggio C., Nor S.A.M., Vaseeharan B. (2021). Interactive effects of freshwater acidification and selenium pollution on biochemical changes and neurotoxicity in Oreochromis mossambicus. Comp. Biochem. Physiol. Part C Toxicol. Pharmacol..

[31] Hou C., Xiao G., Amakye W.K., Sun J., Xu Z., Ren J. (2021). Guidelines for purine extraction and determination in foods. Food Front..

[32] Li X., Liu Z., Li Z., Xiong X., Zhang X., Yang C., Zhao L., Zhao R. (2024). A simple, rapid and sensitive HILIC LC-MS/MS method for simultaneous determination of 16 purine metabolites in plasma and urine. Talanta.

[33] Dehlin M., Jacobsson L., Roddy E. (2020). Global epidemiology of gout: Prevalence, incidence, treatment patterns and risk factors. Nat. Rev. Rheumatol..

[34] Liang J., Wang Z., Zhou L., Niu Y., Yuan C., Tian Y., Takaki K. (2023). Ultrastructural and biochemical changes of sarcoplasmic reticulum in spotted mackerel (Scomber australasicus Cuvier, 1832) muscle during cold storage at 5 °C. Int. J. Food Sci. Technol..

[35] Wu G., Liao J., Zhu X., Zhang Y., Lin Y., Zeng Y., Zhao J., Zhang J., Yao T., Shen X. (2024). Shexiang Baoxin Pill enriches *Lactobacillus* to regulate purine metabolism in patients with stable coronary artery disease. Phytomedicine.

[36] Sadeghi S., Khodanazary A., Hosseini S.M. (2021). The influence of chitosan-carboxymethyl cellulose composite and bi-layer film and coatings on flavor quality and volatile profile of Asian sea bass during storage at refrigerator. J. Food Meas. Charact..

[37] Huang C., Zheng M., Huang Y., Cai L., Zou X., Yao T., Xie X., Yang B., Xiao S., Ma J. (2024). Unraveling genetic underpinnings of purine content in pork. J. Integr. Agric..

[38] Zhou X., Liu K., Shi C., Zhang M., Liu S., Hou C., Di B. (2024). Estimation of the spatial pattern of gout prevalence across China by wastewater-based epidemiology. Sci. Total Environ..

